# Quartz Dust Exposure Affects NLRP3 Inflammasome Activation and Plasma Levels of IL-18 and IL-1Ra in Iron Foundry Workers

**DOI:** 10.1155/2020/8490908

**Published:** 2020-01-07

**Authors:** Alexander Hedbrant, Lena Andersson, Ing-Liss Bryngelsson, Daniel Eklund, Håkan Westberg, Eva Särndahl, Alexander Persson

**Affiliations:** ^1^Department of Medical Sciences, School of Medicine and Health, Örebro University, SE-701 82 Örebro, Sweden; ^2^Inflammatory Response and Infection Susceptibility Centre (iRiSC), Örebro University, SE-701 82 Örebro, Sweden; ^3^Department of Occupational and Environmental Medicine, Örebro University Hospital, SE-701 85 Örebro, Sweden

## Abstract

**Purpose:**

To study the association between inhalation of particulate matter or quartz in Swedish iron foundries and the effects on NLRP3 inflammasome activation.

**Methods:**

Particle exposure measurements were performed during an eight-hour work day for 85 foundry workers at three Swedish iron foundries. Personal sampling was used for measurement of respirable quartz and dust and stationary measurements to obtain exposure measurements for inhalable dust and PM_10_. The NLRP3 inflammasome markers, interleukin- (IL-) 1*β* and IL-18, and inhibitors IL-1 receptor antagonist (IL-1Ra) and IL-18 binding protein (IL-18BP) were measured in plasma. Inflammasome activation was measured by caspase-1 enzymatic activity in monocytes in whole blood by flow cytometry, and expression of inflammasome-related genes was quantified using real-time PCR. Multiple linear regression analysis was used to investigate associations between PM exposures and inflammatory markers. Sex, age, smoking, current infection, BMI, and single nucleotide polymorphism in the inflammasome regulating genes *CARD8* (C10X) and *NLRP3* (Q705K) were included as covariates.

**Results:**

The average exposure levels of respirable dust and quartz were 0.85 and 0.052 mg/m^3^, respectively. A significant exposure-response was found for respirable dust and IL-18 and for inhalable dust and IL-1Ra. Whole blood, drawn from study participants, was stimulated *ex vivo* with inflammasome priming stimuli LPS or Pam3CSK4, resulting in a 47% and 49% increase in caspase-1 enzymatic activity in monocytes. This increase in caspase-1 activity was significantly attenuated in the higher exposure groups for most PM exposure measures.

**Conclusions:**

The results indicate that exposure levels of PM in the iron foundry environment can affect the NLRP3 inflammasome and systemic inflammation.

## 1. Introduction

Exposure to particulate matter (PM) poses a major human health risk. PM exposure in the general environment measured as PM_2.5_ was estimated to contribute to 4.2-8.9 million deaths worldwide in year 2015, predominantly due to cardiovascular diseases (CVD) [1, 2]. PM exposure is monitored based on the aerodynamic size of the particles, which determines their ability to reach different regions of the respiratory tract. The respirable fraction, measured as PM_2.5_ (<2.5 *μ*m) or PM_5_, can reach the alveoli which is the fraction used for occupational limits to quartz exposure. In addition, the thoracic fraction, measured as PM_10_, constitutes PM that can reach beyond the larynx and the inhalable fraction constitutes all PM inhaled through nose and mouth [3]. The iron foundry environment contains high levels of airborne PM and, in addition, the PM contains a high degree of quartz, a well-known hazard for respiratory diseases including silicosis and lung cancer. However, due to current occupational exposure limits to quartz, respiratory diseases such as silicosis are rare [4]. Recent studies instead highlight an increased risk of cardiovascular diseases (CVD) at exposures below current occupational exposure limits [5, 6]. Occupational exposure to quartz is common, with 3 million workers exposed in the European Union [7]. Risk environments include foundry work, mining, rock drilling, stonecutting, tunneling, ceramic manufacturing, and construction work.

Adverse health effects due to particle and quartz exposure are mainly considered to be a result of inflammatory responses at the sites of particle deposition. As such, the occupational limit to quartz is set for the respirable fraction because accumulation of quartz particles in the alveoli is considered the main health concern. The mechanism of particle exposure and systemic inflammation driving diseases including CVD is however not fully understood. One suggested model is that pulmonary inflammation and oxidative stress “spills over” into the blood to cause systemic inflammation, which in turn can contribute to the development of disease [8]. Small particles such as nanoparticles could also penetrate into the bloodstream and directly interact with circulating cells and endothelium thus mediating inflammatory activation in tissues far from the site of particle deposition. Particle-induced inflammation could in part be mediated by the NLRP3 inflammasome and its downstream cytokines, and dusts that contain quartz particles are of extra interest as these are known to activate the NLRP3 inflammasome [9, 10].

The NLRP3 inflammasome is a multiprotein complex that consists of three main proteins, namely, the receptor NLRP3, the adaptor protein PYCARD (also known as ASC), and the enzyme caspase-1 [11]. Upon activation, the inflammasome can cleave the proforms of the cytokine interleukin- (IL-) 1*β* and IL-18 into their active forms.

In general, the NLRP3 inflammasome needs two signals to be activated: first a priming signal, e.g., by endotoxin, which initiates upregulation of NLRP3 and the proforms of IL-1*β* and IL-18, via the transcription factor NF-*κ*B [12]. A second signal (i.e., signal 2) is then needed to initiate the assembly of the inflammasome complex, which in turn causes autoproteolytic activation of caspase-1. In addition to signal 2, which induces formation of the inflammasome complex, activation of NLRP3 is also influenced by genetic factors. For instance, the single nucleotide polymorphism (SNP) Q705K in the *NLRP3* gene is a gain of function polymorphism that can lead to an excessive production of IL-1*β* [13]. The protein CARD8 binds to NLRP3 and prevents inflammasome assembly [14], and the C10X SNP in the *CARD8* gene produce a truncated protein leading to a loss of function for the protein resulting in a lower threshold for inflammasome activation. Both polymorphisms, Q705K and C10X, have been implicated in CVD and other clinically relevant diseases [15–17]. Following inflammasome activation *per se*, inflammasome responses are further regulated by the presence of endogenous inhibitors of the inflammasome-generated cytokines IL-1*β* and IL-18, namely, IL-1 receptor antagonist (IL-1Ra) and IL-18 binding protein (IL-18BP), respectively.

With this background, the aim of this study was to investigate acute inflammatory responses to quartz dust exposure in the iron foundry environment, in particular related to activation or regulation of the NLRP3 inflammasome. This could contribute to a better understanding of the mechanism regarding particle exposure and inflammatory-driven diseases including CVD.

## 2. Methods

### 2.1. Study Group and Iron Foundry

A description of the study group and the iron foundries has been reported previously [18]. In short, the study included 85 foundry workers employed at three Swedish iron foundries with castings mainly based on iron and gray iron alloys, with products such as large details for the wind power industry, motor heads and blocks for trucks, and custom orders. Both mechanized and manual molding and casting occurred at the foundries. Job titles included in the study were sand preparation, melting, core making, molding, casting, shake out, fettling/blasting, inspection, maintenance, transport, and others. Descriptive statistics of the participants is found in Table 1.

### 2.2. Study Design

The sampling was performed between March 2015 and September 2016 at six separate occasions with three, two and one occasions per iron foundry, respectively. Blood and aerosol sampling was performed at the second or third working day after a work-free weekend, with blood drawn in the afternoon (3-4:30 p.m.) after an eight-hour work shift. A questionnaire, addressing questions, which could affect the measured biomarkers, such as height and weight, age, gender, smoking habits, chronic disease, medication, working conditions, and infections, was included for each participant. Chronic diseases were reported for seven workers and included thyroid imbalance, diabetes type 2, scoliosis, hypertension, depression, asthma, and chronic obstructive pulmonary disease.

### 2.3. Aerosol Measurements and Exposure Measures

An extensive description of the dust and quartz exposure measurements and of the analysis has been described previously [18]. In short, 8-hour time-weighted average (TWA) exposures to respirable dust and quartz were measured by personal sampling. Stationary measurements of inhalable dust and PM_10_ (thoracic fraction) were sampled at different departments where the workers were present during the work shift and used as proxies to calculate exposures of these dust fractions. For 28 participants, respirators were used. If a respirator was used, adjusted personal exposure measurements of respirable dust and quartz as well as stationary measurements of inhalable dust and PM_10_ were used as exposure measures.

### 2.4. Caspase-1 Activity

Caspase-1 activity was analyzed in monocytes in whole blood by flow cytometry using the FAM-FLICA Caspase-1 Assay Kit (Immunochemistry Technologies, Bloomington, MN). Directly after a full workday, venous blood was drawn in EDTA tubes (BD Biosciences, San Jose, CA) from the foundry workers. Within 2 hours, 150 *μ*l blood was mixed with an equal volume of RPMI medium supplemented with 10% FBS, HEPES, and penicillin/streptomycin (all from Invitrogen, Carlsbad, CA, USA), with or without Toll-like receptor (TLR) stimuli, 1 ng/ml lipopolysaccharide (LPS, Sigma-Aldrich, St. Louis, MO), or 1 *μ*g/ml Pam3CSK4 (InvivoGen, San Diego, CA). Cells were incubated at 37°C with continuous rotation of the sample tubes. After 3 hours, FLICA substrate was added, with or without additional stimuli with 500 *μ*M ATP and samples were incubated for an additional 1 hour. Thereafter, 100 *μ*l cell suspension was stained with 5 *μ*l CD14-APC antibody (Cytognos, Salamanca, Spain) for 30 min at room temperature before erythrocytes were lysed with 1 ml EasyLyse (Agilent Technologies, Santa Clara, CA) for 15 min at room temperature. Next, the cells were washed and fixed before analyzed on a BD Accuri C6 flow cytometer (BD Biosciences). Monocytes were gated from a CD14/side scatter plot, and 1500 cells were obtained to calculate the median fluorescent signal.

### 2.5. Cytokine Concentration Measurements

IL-1*β* in plasma was analyzed in duplicates on the QuickPlex SQ120 instrument (MesoScale Diagnostics, Rockville, MD) using their IL-1*β* V-Plex assay according to the manufacturer's instructions. ELISA was used to measure IL-18 (DuoSet ELISA), IL-18BP (Quantikine ELISA), and IL-1Ra (Quantikine ELISA) according to the manufacturer's instructions (R&D Systems, Minneapolis, MN).

### 2.6. Gene Expression Analysis

Whole blood was collected in PAX-gene blood RNA tubes (Qiagen, Hilden, Germany) from workers after a full workday. RNA was isolated using the PAXgene Blood RNA Kit (Qiagen) according to manufacturer's instructions. RNA quantity and quality was assessed on a Bioanalyzer 2100 instrument with the RNA 6000 Nano Kit (Agilent Technologies) and the lowest RNA integrity number (RIN) was 7.9. cDNA was transcribed from 7.5 ng RNA/*μ*l cDNA reaction volume using the High-Capacity Reverse Transcription Kit (Thermo Fisher, Waltham, MA) according to the manufacturer's instructions. Real-time PCR was performed in duplicates using the PowerUp SYBR Green Master Mix (Thermo Fisher) in 384 well format in duplicates with 1.9 ng RNA equivalents of cDNA, 300 nM primers in a 10 *μ*l reaction volume on the QuantStudio 7 Flex Real-Time PCR instrument (Thermo Fisher). Mastermix and samples were pipetted by a pipetting robot (PIRO, Dornier LabTech Systems, Lindau, Germany). PCR product specificity of each primer pair was confirmed by melt curve analysis and also by agarose gel electrophoresis of the PCR products. For a standard curve, peripheral blood mononuclear cell (PBMC) was stimulated with 1 *μ*g/ml ultrapure LPS (InvivoGen) for 48 h prior to extraction of RNA using the QIAamp RNA Mini Kit (Qiagen) and reverse transcribed to cDNA using the high-capacity reverse transcription cDNA Kit (Thermo Fisher). A 6-point standard curve with a 1 : 4 dilution was run in duplicates for each gene target. All primer pairs had an efficiency of at least 90% calculated from the standard curves. *HPRT1* was chosen as the best reference gene out of four targets (*HPRT1*, *PPIB*, *RPLP1*, and *TBP*) based on the NormFinder algorithm [19]. For duplicates with a CV above 16.0%, the samples were rerun until a CV lower than 16.0% was achieved. The lowest point of the standard curve was given the arbitrary value of 1 and each following point of the standard curve multiplied by 4. From the standard curve, linear regression was used to calculate a value for each sample and target gene, which was divided with the value obtained for the reference gene (normalized value). The normalized value was used for statistical analysis to evaluate effects of exposure. All primer sequences except for *IL18BP* were collected from the primer bank database [20]. All primer pair products except for *PYCARD* span at least one intron. The primer sequences are presented in Table 2.

### 2.7. Single Nucleotide Polymorphism Analysis

DNA was extracted from dried blood spots on Whatman FTA cards (GE Healthcare, Chicago, IL) or from whole blood. DNA from dried blood spots and from whole blood was extracted using the QIAamp DNA Mini Kit (Qiagen) and the NucleoSpin Blood Kit (Macherey-Nagel, Düren, Germany), respectively, according to the manufacturer's instructions. Single nucleotide polymorphism (SNP) analysis was performed with TaqMan assays (Thermo Scientific) for *CARD8* C10X (rs2043211) and *NLRP3* Q705K (rs35829419) using the TaqMan Genotyping Master Mix (Thermo Scientific). DNA extracted from dried blood spots was used for SNP genotyping, and 14 DNA samples extracted from whole blood were used to confirm results of the C10X analysis.

### 2.8. Statistical Analysis

**Figure 1:**
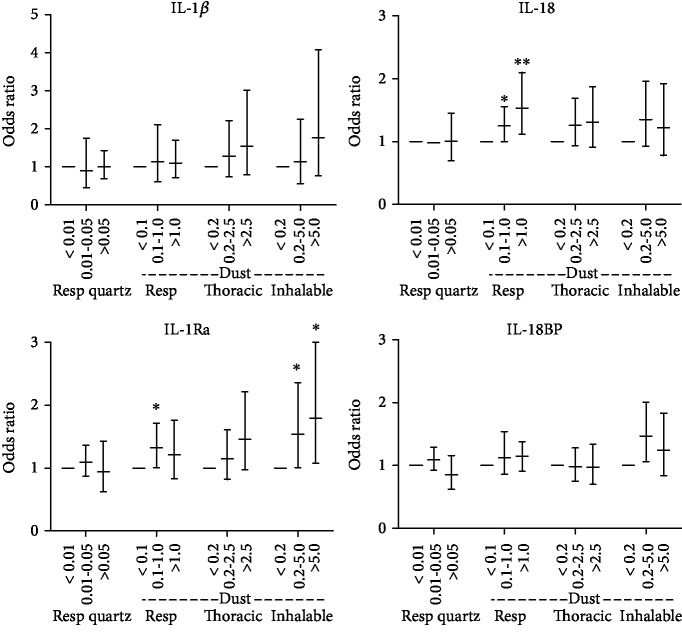
Multiple linear regression for plasma concentration of indicated cytokines, associated to exposure levels (mg/m^3^) of indicated particle exposure. Error bars show 95% CI. ^∗^*p* < 0.05, ^∗∗^*p* < 0.01.

Study participant characteristics are presented using descriptive statistics (Figure 1). Multiple linear regression was used to describe the exposure-response relationship to PM or quartz and inflammatory markers, dividing each exposure measure into three classes (high, middle, and low exposure, with low exposure set as reference). The high-exposure groups for respirable quartz, respirable dust, inhalable dust, and thoracic dust were set to >0.05, >1.0, >5, and >2.5 mg/m^3^, respectively, corresponding to approximately half of the current occupational exposure limits for Sweden, where the participating foundries are located. The exposure classes had a minimum of 8 workers in a group and are summarized in Table 3.

The log-transformed values were used for cytokine concentration, mRNA expression, or caspase-1 activity. The odds ratio (OR) and 95% confidence interval (CI) presented are the antilog values. Covariates included (1) age (dichotomized by median), (2) smoking (current, never, and ex-smoker), (3) gender, (4) current infection, (5) C10X CARD8 polymorphism (wild-type (wt) or combined homozygous or heterozygous C10X), and (6) Q705K NLRP3 SNP (wt or heterozygous Q705K (no homozygote present)). Results are displayed as OR with 95% CI and *p* < 0.05 is used for statistical significance.

### 2.9. Ethics

Informed consent was obtained for all individual participants included in the study. The study was approved by the Regional Ethical Review Board, Uppsala, Sweden, dnr. 2015/066.

## 3. Results

### 3.1. Quartz and Dust Exposure Levels in the Foundry Environments

Exposure measures used in this study included inhalable dust, i.e., all dust inhaled through nose and mouth; thoracic dust (measured as PM_10_, <10 *μ*m aerodynamic size), i.e., the size fraction able to reach beyond the larynx; respirable dust (<5 *μ*m), i.e., the size fraction able to penetrate the alveoli; and respirable quartz, i.e., the quartz content of respirable dust. The 8-hour TWAs for the respirable quartz exposures measured by personal sampling of the 85 participants ranged from 0.001 to 0.61 mg/m^3^, with a mean of 0.052 mg/m^3^, and the corresponding respirable dust levels varied between 0.065 and 9.7 mg/m^3^. The inhalable dust levels ranged from 0.045 to 16 mg/m^3^, and the PM_10_ levels from 0.078 to 16 mg/m^3^ with a mean of 3.4 mg/m^3^.

The complete exposure levels to quartz and PM of various size fractions in the studied iron foundries, measured both by personal and stationary monitoring, have been published elsewhere [18]. For the personal measurements of the 28 persons using respirators, we calculated adjusted background respirable dust and quartz concentrations to use as our exposure measures for all exposure-response analyses.

### 3.2. Cytokine Levels

The plasma concentration of measured cytokines (IL-1*β*, IL-18, IL-1Ra, and IL-18BP) is found in Table 4, and multiple linear regression analysis of cytokine levels and quartz and dust exposure is found in Figure 1. Exposure to inhalable dust was significantly associated with increased IL-1Ra plasma levels for the middle-exposure (OR = 1.54, *p* = 0.047) and high-exposure (OR = 1.79, *p* = 0.027) groups compared to the low-exposure group. Thoracic dust showed a similar nonsignificant trend for IL-1Ra with higher protein levels with increasing exposure. Exposure to respirable dust was significantly associated with higher IL-18 plasma levels for the middle-exposure (OR = 1.25, *p* = 0.046) and high-exposure (OR = 1.53, *p* = 0.009) groups compared to the low-exposure group. Quartz exposure, measured as respirable quartz, was not significantly associated with any of the measured cytokines. Regarding the included covariates, for one or more exposure measures, age was a significant covariate for IL-18 (OR = 1.01), BMI for IL-1Ra (OR = 1.06) and IL-1*β* (OR = 1.04), smoking for IL-18BP (OR = 1.22), and *CARD8* C10X SNP for IL-1*β* (OR = 1.42).

### 3.3. NLRP3 Inflammasome Activation

Blood drawn from the workers after shift was exposed to inflammasome priming and activating chemicals to assess the responsiveness of the NLRP3 inflammasome, measured as caspase-1 enzymatic activity in monocytes compared to unstimulated control. The stimuli LPS and Pam3CSK4 are mainly considered to affect inflammasome priming, whereas ATP triggers inflammasome activation in primed cells. The priming agents LPS or Pam3CSK4 alone gave a mean 47% and 49% increase in caspase-1 enzymatic activity in monocytes, respectively, compared to unstimulated control. NLRP3 inflammasome activation, due to ATP stimulation, alone or in combination with LPS priming, gave a mean increase of 20% or 59%, respectively, compared to unstimulated control (Table 4).

**Figure 2:**
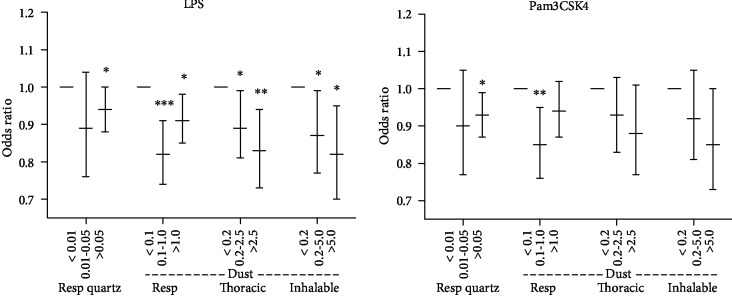
Multiple linear regression for increase in caspare-1 enzymatic activity in monocytes following LPS or Pam3CSK4 stimulation associated to exposure levels (mg/m^3^) of indicated particle exposure. Error bars show 95% CI. ^∗^*p* < 0.05, ^∗∗^*p* < 0.01, ^∗∗∗^*p* < 0.001.

Multiple linear regression analysis of dust and quartz exposure and caspase-1 activity is shown in Figure 2. The middle- and high PM-exposure groups were significantly associated with a lower increase in caspase-1 activity in LPS-stimulated cells, measured as percent increase to unstimulated cells, compared to the low-exposure group for all dust fractions (inhalable, thoracic, respirable). This was also found for respirable quartz for the highest exposure group. The same trends were obtained using Pam3CSK4, another priming stimuli, for all exposure measures, however, only significant for the highest exposure groups for respirable quartz and inhalable dust. ATP treatment, with or without LPS priming, was not significantly associated with any of the exposure measures (data not shown). Regarding covariates and the caspase-1 activity measurements, *NLRP3* Q705K SNP was significant for the Pam3CSK4 treatment (OR = 0.9).

### 3.4. Transcription of NLRP3 Inflammasome and Inflammatory Genes in Whole Blood

**Figure 3:**
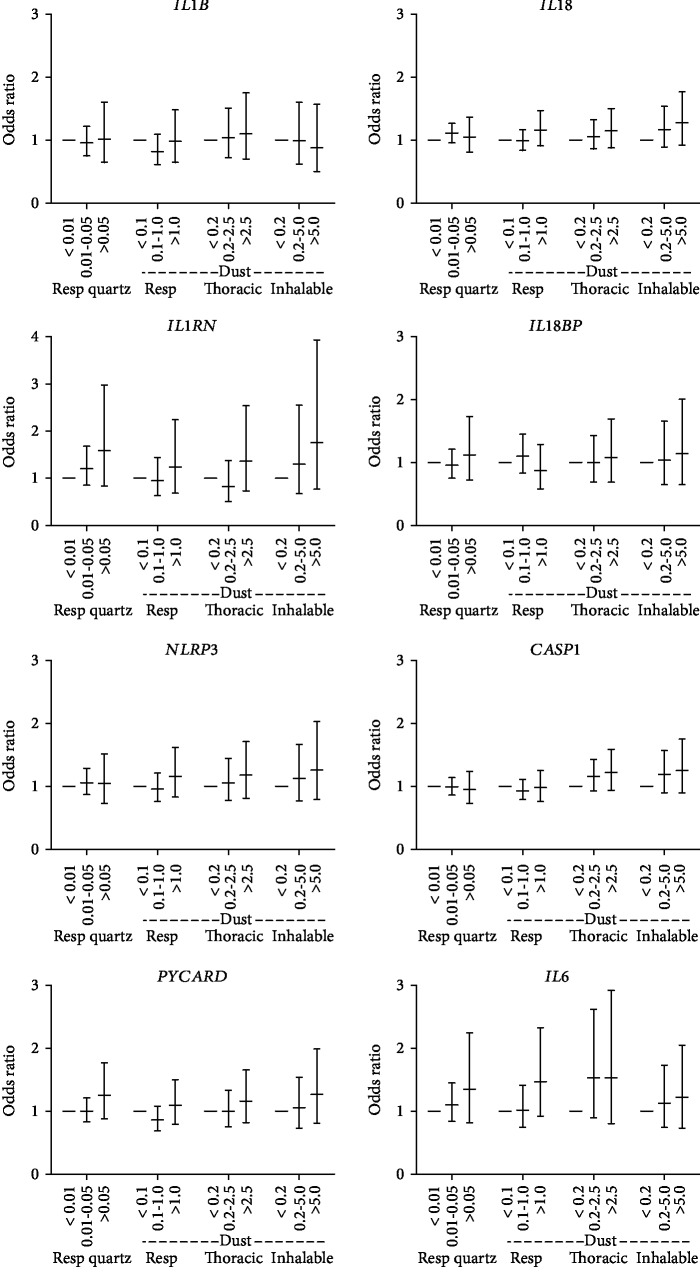
Multiple linear regression for indicated mRNA levels in whole blood associated to exposure levels (mg/m^3^) of indicated particle exposure (no significant changes). Error bars show 95% CI.

The mRNA expressions analyzed included the inflammasome components *PYCARD* and *CASP1*, the priming or NF-*κ*B-dependent targets *NLRP3*, *IL1Β*, *IL18*, and *IL6*, and the inhibitors *IL1RN* and *IL18BP*. Whole blood mRNA expression levels for all measured gene targets are shown in Table 4. No significant exposure-response association was found for any of the studied gene transcripts (Figure 3). Nonsignificant trends indicate increased gene expression with higher PM exposures for some gene transcripts, including *IL1RN* and *IL18*, in accordance with the results on plasma protein concentration for these targets. The same trends were observed for the priming/NF-*κ*B-regulated genes *NLRP3* and *IL6* and for the inflammasome component *PYCARD*.

## 4. Discussion

One of the main findings of this study is the exposure-response association for exposure to foundry dust and plasma levels of IL-18 and IL-1Ra. The NLRP3 inflammasome, which processes IL-1*β* and IL-18 to its active forms, can be activated by particulate matter, including quartz, and our results indicate that this occurs at exposure levels present in the foundry environment. Interestingly, different dust size fractions were associated with IL-18 and IL-1Ra levels, respectively. IL-18 was associated with respirable dust, i.e., particles that deposit in the alveoli, whereas IL-1Ra was more strongly associated with the inhalable fraction, i.e., particles that are mainly deposited in the upper airways. IL-1Ra is found in high levels (ng/ml) in the fluids from upper airways, including nasal lavage, nasal secretion, and saliva [21–23]. Therefore, IL-1Ra is likely an important mediator in the upper airways to prevent exaggerated inflammatory responses.

We did not observe a statistically significant exposure-response for IL-1*β*; however, reliable quantification of IL-1*β* is challenging, as plasma levels are very low. Instead, the inhibitor IL-1Ra, which is more easily quantifiable, has been suggested to reflect higher IL-1*β* activity [24]. BMI was a significant covariate for IL-1Ra in this study, in line with previous studies demonstrating that IL-1Ra is elevated in individuals with cardiometabolic risk factors including obesity and type 2 diabetes mellitus [24, 25].

We did not observe any statistical differences in mRNA expression of *IL18* or *IL1RN* in whole blood following PM exposure. The discrepancy between protein and mRNA data could be due to cytokine production at the site of exposure in the respiratory tract, rather than being produced by circulating leukocytes, as is a suggested model for CVD and particle exposure [8]. However, as mentioned below, NLRP3 inflammasome activation in circulating monocytes appears to be affected by PM exposure; therefore, a possible contribution from circulating cells should not be excluded.

The other main finding of our study was an observed exposure-response association for quartz and PM exposure and NLRP3 inflammasome responsiveness to activating signals *ex vivo*. Caspase-1 enzymatic activity was measured as a marker of inflammasome activation. The analysis showed that with higher exposure to particle fractions, monocytes show less responsiveness to LPS and Pam3CSK4 stimulation, as measured by capsase-1 activity. The low responsiveness could indicate that higher exposure groups have a preactivated inflammasome, rendering them less responsive to additional stimulation.

Pam3CSK4, in contrast to our study, has been shown to affect inflammasome priming but not activation [26]. As such, either there are other inflammasome activating compounds present in the measured blood samples, or we measure some unspecific protease activation of the caspase-1 probe.

Inflammasome priming causes an upregulated gene expression of *IL1Β*, *IL18*, and *NLRP3* through NF-*κ*B activation. To determine if quartz or PM exposure in the foundry environment affect inflammasome priming, the mRNA expression level of genes upregulated by inflammasome priming or NF-*κ*B activation was analyzed in whole blood. Nonsignificant trends of higher gene expression of *NLRP3*, *IL6*, and *IL18* were observed indicating that PM exposure may affect priming in monocytes.

The SNPs Q705K and C10X were included as covariates as they both have been shown to increase NLRP3 inflammasome activation and IL-1*β* production. In accordance with previous reports, C10X was positively associated with IL-1*β* levels in this study and Q705K was associated with caspase-1 enzymatic activity.

The inflammatory responses to dust quartz exposure found in this study could contribute to our understanding of the health risks associated with working in the foundry environment. The foundry environment with current exposure levels to quartz dust has been associated with an increased risk of CVD [27, 28]. Inflammation contributes to CVD pathology, and the NLRP3 inflammasome may play an important role in CVD development [29, 30] and other inflammatory-mediated diseases [31]. Importantly, plasma levels of the cytokines IL-18 and IL-1Ra that demonstrated a positive association with particle exposure in this study have both been associated with CVD risk [32–34]. Interestingly, our results demonstrate stronger associations with the inflammasome-related cytokines and PM exposure measures, including respirable dust, compared to respirable quartz. This is somewhat unexpected since quartz particles are known to mediate NLRP3 inflammasome activation demonstrated *in vitro* and in rodent models *in vivo* [10]. In our previous paper regarding inflammatory and coagulatory markers from the same sample cohort, we also found significant associations for some biomarkers and different PM fractions but no significant associations to respirable quartz [18]. This implicates that the dusty work environment, rather than present quartz exposures, could be a risk factor for activation of inflammation in the foundries. As such, in order to protect worker's health, it could be more valuable to lower occupational limits to dust exposure, i.e., respirable dust, rather than quartz. However, this is something that needs to be studied further, also in other work environments.

There are some limitations to this study. The sampling was performed on the second or third day following a work-free weekend; therefore, it is possible that exposures from previous work days obscure results. In addition, individuals exposed highly during the measuring day are likely to be highly exposed in general. Therefore, there is a risk that we do not measure acute daily differences, but an effect of accumulated exposure. However, studies in the general environment have observed increased risk of CVD due to short-term variations in PM exposure, indicating that daily variations can have biological effects [35]. Furthermore, the iron foundry environment contains, in addition to PM and quartz, chemical agents from the binders used together with the sand to form the molds. Exposures to furfuryl alcohol and formaldehyde have been observed, particularly in the molding and casting areas [36, 37]. We do not have data on the study participant's exposures to chemical agents such as furfuryl alcohol or formaldehyde due to use of chemical binders during the sampling days and cannot exclude confounding effects due to such exposures. Another limitation is the use of respirators for some of the study participants. Although we compensate for the use of respirator, the actual exposure is still more unreliable, which depends on correct use of the respirator and during which work tasks the respirator was used.

In conclusion, we found exposure-response association with PM exposure and markers of NLRP3 inflammasome activation, including IL-18 and IL-1Ra plasma levels, and effects on inflammasome activation in circulating monocytes for various PM and respirable quartz exposure. These results indicate that PM exposure in iron foundries may affect NLRP3 inflammasome signaling and initiate inflammatory processes. This could have implications for development of inflammatory-driven diseases including CVD.

## Figures and Tables

**Table 1:** Study participant characteristics.

Participants (*n* = 85)			
Gender	Male	Female	
*n*	80	5	
Age (years)	≤44	≥45	
*n*	44	41	
BMI	<26.8	>26.8	
*n*	42	43	
Smoking (2 missing values)	Current smoker	Ex-smoker	Never-smoker
*n*	17	18	48
NLRP3 Q705K	Wild type	Heterozygote	Homozygote
*n*	74	11	0
CARD8 C10X	Wild type	Heterozygote	Homozygote
*n*	33	41	11
Ongoing infection	Yes	No	
*n*	7	78	

**Table 2:** PCR primers.

Gene symbol	Forward primer 5′-3′	Reverse primer 5′-3′
IL1Β	ATGATGGCTTATTACAGTGGCAA	GTCGGAGATTCGTAGCTGGA
IL1RN	CATTGAGCCTCATGCTCTGTT	CACTGTCTGAGCGGATGAA
IL6	CCTGAACCTTCCAAAGATGGC	TTCACCAGGCAAGTCTCCTCA
IL18	TCTTCATTGACCAAGGAAATCGG	TCCGGGGTGCATTATCTCTAC
IL18BP	AGCAGCTAAGCAGTGTCCAG	TGCAGGCCACACAGGATAAG
NLRP3	GATCTTCGCTGCGATCAACAG	CGTGCATTATCTGAACCCCAC
CASP1	TTTCCGCAAGGTTCGATTTTCA	GGCATCTGCGCTCTACCATC
PYCARD	TGGATGCTCTGTACGGGAAG	CCAGGCTGGTGTGAAACTGAA
HPRT1	CCTGGCGTCGTGATTAGTGAT	AGACGTTCAGTCCTGTCCATAA
PPIB	AAGTCACCGTCAAGGTGTATTTT	TGCTGTTTTTGTAGCCAAATCCT
RPLP0	GCAGCATCTACAACCCTGAAG	CACTGGCAACATTGCGGAC
TBP	CCCGAAACGCCGAATATAATCC	AATCAGTGCCGTGGTTCGTG

**Table 3:** Calculated exposure levels to different particle exposure measures for the participating iron foundry workers.

Exposure class	Exposure level (mg/m^3^)		
	Low	Middle	High
Respirable quartz	<0.01	0.01-0.05	>0.05
*n*	41	36	8
Respirable dust	<0.1	0.1-1.0	>1.0
*n*	16	58	11
Inhalable dust	<0.2	0.2-5.0	>5.0
*n*	10	65	10
Thoracic dust	<0.2	0.2-2.5	>2.5
*n*	14	54	17

**Table 4:** Descriptive statistics of cytokine, mRNA, and caspase-1 data.

Sampling	*n*	Mean	Median	Min	Max
Cytokines		Plasma concentration (pg/ml)			
IL-1*β*	85	0.032	0.027	0.001	0.107
IL-18	85	482	439	219	1328
IL-1Ra	85	271	193	82	1490
IL-18BP	85	2348	2162	932	5954
mRNA expression		Normalized expression, arbitrary unit			
IL1Β	84	0.07	0.06	0.02	0.15
IL18	84	1.40	1.29	0.77	2.74
IL1RN	84	0.74	0.64	0.15	2.52
IL18BP	84	0.96	0.93	0.19	1.88
NLRP3	84	0.56	0.53	0.15	1.42
PYCARD	84	8.38	8.26	2.74	18.31
CASP1	84	0.94	0.89	0.52	1.71
IL6	84	0.004	0.004	0.001	0.010
Caspase-1 enzymatic activity in monocytes		% of unstimulated control			
Unstimulated		100	100	100	100
LPS	75	147	149	109	191
Pam3CSK4	75	151	153	115	184
ATP	35	120	119	61	241
LPS+ATP	35	159	157	89	250

## Data Availability

Previously reported PM exposure measurement data were used to support this study and are available at [10.1007/s00420-019-01446-z]. This prior study is cited at relevant places within the text as references [18]. The datasets generated during and/or analyzed during the current study are available from the corresponding author upon reasonable request.
